# Characterisation of Polyamide (PA)12 Nanocomposites with Montmorillonite (MMT) Filler Clay Used for the Incremental Forming of Sheets

**DOI:** 10.3390/polym11081248

**Published:** 2019-07-28

**Authors:** Andrej Borić, Alena Kalendová, Michal Urbanek, Tomaž Pepelnjak

**Affiliations:** 1 Faculty of Engineering, University of Rijeka, Vukovarska 58, 51 000 Rijeka, Croatia; 2 Faculty of Technology, Tomas Bata University in Zlin, Vavrečkova 275, 760 01 Zlin, Czech Republic; 3 Centre of Polymer Systems, University Institute, Tomas Bata University in Zlin, Trida Tomase Bati 5678, 760 01 Zlin, Czech Republic; 4 Faculty of Mechanical Engineering, University of Ljubljana, Aškerčeva 6, 1000 Ljubljana, Slovenia

**Keywords:** polymer, nanocomposite, polyamide, PA12, clay, Cloisite 93A, morphology, mechanical properties

## Abstract

In this paper, the preparation and characterisation of polymer materials suitable for single point incremental forming (SPIF) technology were performed. Three different kinds of mixtures were selected: a mixture of neat polyamide 12 (PA12), a nanocomposite with PA12 matrix and 1% clay (Cloisite 93A), and a nanocomposite with PA12 matrix and 3% clay (Cloisite 93A). Materials were produced using a melt intercalation method followed by compression moulding. According to the needs of SPIF technology, morphological and mechanical properties were investigated in the obtained mixtures. Transmission electron microscopy (TEM) and X-ray diffraction (XRD) were used to characterize morphological properties. It was determined that the most desired obtained exfoliated structure of clay in the polymer matrix was achieved. Static tensile testing and dynamic mechanical analysis as well as the determination of glass transition temperature and crystallinity of all analysed materials were used to obtain mechanical and thermal properties of the mixtures. The results obtained for each mixture were compared with respect to the content of clay. The content of clay (Cloisite 93A) showed a strong influence on the properties of the obtained materials. The presence of clay (Cloisite 93A) affected the increase of tensile strength and Young’s modulus, while its influence on the attained elongation was not unique.

## 1. Introduction

Polyamides (PA) are polymers that contain repeating units linked with amide bonds. They are indicated by the number of carbon atoms in the reacting monomers. Polyamides are widely used materials due to their tuneable properties and acceptable prices [1]. Layered silicates (so-called clays) are the most studied class of nanoscale fillers because they can improve many material properties [2]. Their addition to existing materials can be performed at a relatively low cost. Studies have shown that nanocomposites that consist of polymer and layered silicate have significantly improved properties when compared to neat polymer or conventional composites at both macro- and micro-scales [3,4]. To date, global research activities have confirmed improvements in the following material properties: mechanical properties [5,6,7,8], flame retardancy [9,10,11,12,13,14,15], barrier properties [16,17,18,19,20,21,22,23,24], ionic conductivity [25,26], thermal stability [27,28], and the biodegradability of biodegradable polymers [10,29]. Previous studies on polymer nanocomposites in the case of the PA matrix and clay fillers are mainly based on the use of polyamide 6 (PA6) matrix followed by polyamide 66 (PA66) matrix, while polyamide 12 (PA12) is the least represented of these materials. One of the main reasons for the large number of research composites based on PA6 and clay is certainly the positive results that researchers from Toyota Central Research and Development Labs gained in the late 1980s and early 1990s [20,30,31,32,33,34,35]; in their studies, the properties of the composites based on PA6 and montmorillonite (MMT) filler were obtained via in situ polymerisation. Later research has shown the possibility of obtaining a nanocomposite based on PA6 and clay by melt intercalation [5,36,37]. This method could be preferable for preparing nanocomposites for commercial purposes [38]. Composites based on PA12 matrix and clay filler are the least investigated, but as other PAs, it can be prepared by in situ polymerization [39,40] and by melt intercalation [23,41,42,43,44]. Moškova et al. [44] conducted research on nanocomposites preparation based on a PA12 matrix and MMT fillers. Prediction of the tendency of organo-modified clays was made using rheological measurements. Follain et al. [23] prepared hybrid films with barrier properties, which are composed of PA12 matrix and MMT filler (Cloisite 30B and Cloisite Na+) using a melt intercalation method. The research of mechanical properties of nanocomposites with clay fillers has shown that clay affects the mechanical properties of nanocomposites by increasing the tensile strength and Young’s modulus while slightly reducing the impact strength. The polymer nanocomposite characterizations and morphology were conducted using transmission electron microscopy (TEM) [45], wide angle X-ray diffraction (WAXD) [45], scanning electron microscopy (SEM) [45], dynamic mechanical analysis (DMA), differential scanning calorimetry (DSC), and X-ray diffraction (XRD) while the rheological measurement are performed using specialized rheometers [46,47,48]. 

PA12 is a polymer that has a wide range of applications, especially in the automotive and electrical industries as well as in mechanical engineering, precision moulding, and sports and leisure goods. In the presented research, PA12 was selected because it has higher tensile modulus compared to PA6 [49]. Also, PA12 has a higher elongation at break than PA6 and PA66. High elongation is preferred because of the way the single point incremental forming (SPIF) method works. Compared to the glass transition temperature *T*_g_, PA12 is also advantageous, since it has a lower *T*_g_ than the other two polyamides [49]. In comparison to other polyamides, PA12 has the lowest amount of water and moisture absorption, thereby facilitating the preparation of polymeric sheets [49]. Furthermore, PA12, which has the smallest water absorption of among all PA groups analysed to date, is advantageous since water-dispersion lubricants are often used during the SPIF. In recent years Cloisite 93A filler has been used as a reinforcing agent in polymer matrices: polyamide 12 [43], polyamide 6 [50,51], polypropylene [52], poly(lactic acid) [53], epoxy [54,55], and polychloroprene [56].

**Figure 1:**
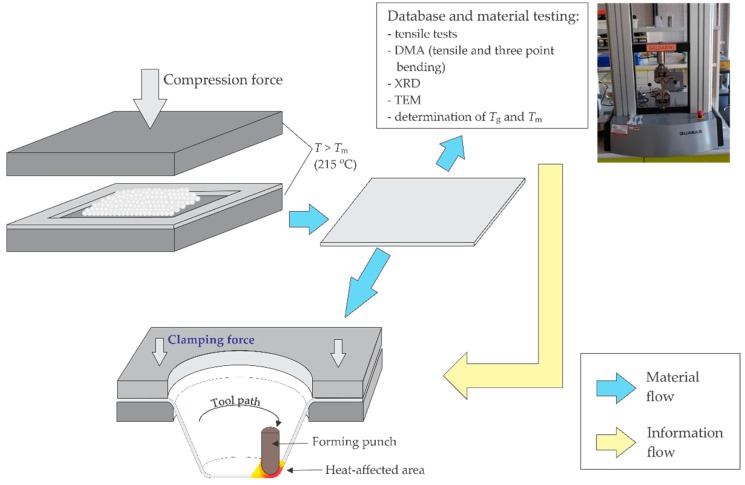
Schematic representation of the preparation of polyamide (PA) 12/clay nanocomposite sheets suitable for the single point incremental forming (SPIF) process.

Analysing applicability of PA for SPIF technology, Martins et al. [57] obtained very high ductility of this polymer group, indicating that it is suitable for parts with large wall angles. Marques [58] et al. conducted good formability of PA during SPIF, in which the twisting of material could be prevented. Therefore, in the presented research, the composite based on PA12 matrix and clay filler were prepared for the needs of the SPIF process (Figure 1). SPIF is a flexible forming process for the manufacturing of components from various materials in the form of sheets or plates. The main advantage of the SPIF process is to produce complex sheet parts in small batches in an economical way. In the beginning, the SPIF process was used on sheet metal [59,60] only; however, in the last ten years, it has also been applied to various types of polymers. As the first application of the SPIF process with polymers, the forming of polyvinylchloride (PVC) by Franzen was acknowledged [61] followed by the SPIF on polypropylene (PP) and other thermoplastics [62,63]. Since the further spread of SPIF applications on polymer materials is expected, the crucial material parameters affecting this process should be analysed. The SPIF process heats the material in the contact area below the tool sliding along the predefined tool path. Together with sliding along the material surface, the tool can also rotate and/or vibrate. Since the material is significantly plastically deformable mainly only above the glass transition temperature *T*_g_, it is necessary to evaluate it in a wide temperature range from room temperature to the temperatures above *T*_g_. Finally, the polymer material also hardens as a result of plastic deformation. Therefore, the morphological, structural, and mechanical properties of the obtained composites need to be examined.

## 2. Materials and Methods 

### 2.1. Materials

In this research, polymer PA12 and clay were used. The PA12 used was the product of EOS GmbH (Krailling, Germany), with the code name PA 2200. The mentioned polymer is white and in powder form with an average grain size of 60 μm [64]. It is primarily used in the additive selective laser-sintering (SLS) process to shape an object [65,66,67,68] with excellent material properties, such as high strength and stiffness, excellent long-term constant behaviour and bio-compatibility [64]. According to the manufacturer’s data [64], this PA12 2200 polymer has the melting temperature at *T*_m_ = 176 °C. The Vicat softening temperature for heating rate 50 °C/h and load 50 N is 163 °C [64]. Bulk density of PA 12 2200 according to EN ISO 60 is 450 kg/m^3^, and the density of laser-sintered material is 930 kg/m^3^ [64,69]. 

The flexural modulus at 23 °C is 1500 MPa, while the reported tensile modulus is between 1650 MPa and 1700 MPa [69]. For the material PA12 2200, the manufacturer reports an attainable 24% strain at break for specimens produced with additive technologies while data for injection-moulded material are not available. Rahim et al. have compared the EOS PA12 2200 samples prepared with injection moulding and additive technologies [70]. They obtained elastic modulus of 1376 MPa for injection-moulded samples and 1037 MPa for 3D-printed samples. In contrast, the storage modulus for injection-moulded samples was 1500 MPa and 1250 MPa for 3D-printed samples. According to Dotchev and Yusoff, the melt flow rate (MFR) for PA 2200 could vary from 50 to 60 g/10 min for fresh powder, depending on selected batches [71]. The nanofiller used was a product of the Southern Clay Products (part of BYK Additives and Instruments, Wesel, Germany), known as Cloisite 93A. This material is a modified natural MMT using a ternary ammonium salt, with a concentration of 90 milliequivalents per 100 g [72]. The spacing between the Cloisite 93A layers is 2.36 nm, according to the manufacturer’s declaration, while the specific gravity is 1.88 g/cm^3^ [72]. 

### 2.2. Preparation

For the purposes of this research, the following mixtures were prepared: neat PA12, PA12 with 1% Cloisite 93A and PA12 with 3% Cloisite 93A. A larger amount of clay would result in a stiffer nanocomposite [43,73], which is more difficult to form using the SPIF process. Prior to the mixing, the PA12 and clay were dried for 24 hours at 60 °C. The granulate was prepared with a Brabender extrusion line (Duisburg, Germany) which was composed of a twin-screw counter-rotating extruder, plasti-corder, conveyor belt and palletizer (Table 1). During the extrusion process, the following technological parameters were selected: the temperature on the first zone was 180 °C, on the second zone 220 °C, and on the third zone 220 °C counting from the feeder to the nozzle while the rotation speed of the screws was 30 rpm. After the extrusion process, the PA wires were chopped into granulate. To attain a good homogeneity and dispersion of Cloisite 93A in the polymer composition, the process of extrusion was repeated three times by the same mixing conditions. Repeating the granulation process was necessary due to the short extruder ratio of screw length *L* versus its diameter *D* of *L*/*D* = 6 only. The longer residence time within the extruder generally favours a better dispersion of the filler [38]. All granulates were dried prior to further processing in order to minimise the amount of their humidity. Thereafter, the sheets were produced by compression moulding into 2 mm thick sheets at a temperature of 215 °C.

### 2.3. Morphological and Structural Characterisation

The morphological properties of the samples were tested with a Malvern Panalytical X’Pert Pro X-ray diffractometer (XRD, Malvern, UK) device. Testing was performed at 40 kV and 30 mA. Absolute scanning mode was selected with a starting angle of 1° and a final angle of 50° with a step of 0.03° and a time of 38 s at each step. The nanoparticle scattering was investigated using transmission electron microscopy (TEM) using a JEOL JEM 2100 device (Tokyo, Japan) with 200 kV acceleration voltage. Ultra-thin sections of material with the thickness of 90 nm were cut from sheets using a Leica EM UC7 ultramicrotome (Wetzlar, Germany). Additional to the XRD and TEM analysis, differential scanning calorimetry (DSC) was used to evaluate the crystallinity of prepared samples. The experiment was performed on a Mettler Toledo DSC 700/1 device (Columbus, OH, USA) in the temperature range from 25 °C to 210 °C, heating rate 20 K/min, and two cycles under nitrogen atmosphere. The weight of sample was in the 5–10 mg range. The evaluation was carried out from the second sample heating.

### 2.4. Mechanical Characterisation

In the case of mechanical tests, static tensile testing and dynamic mechanical analysis (DMA) were performed. 

For the purpose of static tensile testing, a Galdabini Quasar 25 tensile testing machine (Cardano al Campo, Italy) delivering the maximal load of 25 kN was used. Test samples were prepared according to the EN ISO 527-2 standard [74]. For each material mixture, five test samples were made. The tensile testing was performed at room temperature and a speed of 50 mm/min in accordance with EN ISO 527-3 standard [75].

DMA tests were performed with a Mettler Toledo DMA 1 device (Columbus, OH, USA). Two different DMA tests were performed for the purpose of this research: a tensile test and a three-point bending test. Both tests were carried out as a function of temperature.

DMA tensile tests were performed from the initial temperature of 25 °C up to 150 °C at a heating rate of 2 °C/min, and a frequency of 1 Hz with an amplitude of 100 µm. The maximal testing temperature was below the PA12 melting temperature as presented in Section 3.1. Testing was done for all material mixtures. Additionally, if samples are thicker than one millimetre, it is recommended to select the bending mode, since such samples could be too stiff for the tension testing mode [76]. With the analysed thickness of PA and PA-nanocomposite sheets of 2 mm supplementary to DMA tensile test, the three-point bending test was also conducted. This type of test is used for very stiff parts, such as composite materials or thermosets, particularly below the glass transition temperature [77]. DMA three-point bending tests were performed at temperatures from 25 °C to 150 °C at a heating rate of 2 °C/min at a frequency of 1 Hz, 5 Hz, and 10 Hz; all with an amplitude of 100 µm. The selection of frequency range is described in detail.

The critical parameter when planning a DMA test is to select the proper frequency. The most suitable method is to use the frequency similar to the loading conditions later applied in the real application as in the case of the SPIF process [78]. Another way to choose frequency is based on ASTM methods for DMA testing, which contain standards for each industry [78]. The last way of selecting the frequency for DMA testing is random frequency selection, which is very common in research practice; the frequencies usually chosen are 1 Hz or 10 rad/s [78].

**Figure 2:**
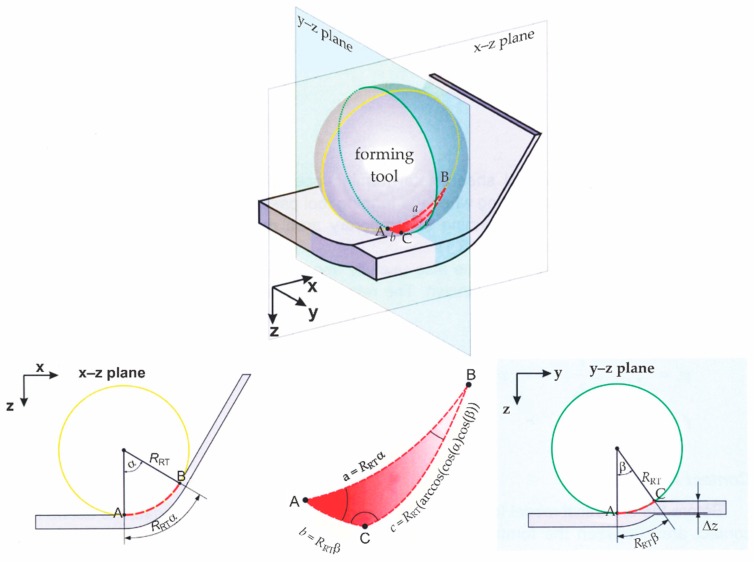
Determination of contact area during the SPIF process using spherical geometry [79].

For the needs of determining the actual frequency that occurs during the SPIF process, the spherical tool geometry and tool path performed in a time unit should be considered. The contact path of the SPIF process determined by Petek [79], as shown in Figure 2, can be represented by Equations (1) and (2):

(1) $$\beta =\cos^{-1}\left(1-\frac{\Delta z}{R_{RT}}\right),$$

(2) $$b=R_{RT}\cdot \beta .$$

The results for the inner angle of spherical triangle *β* obtained from Equation (1) and side of spherical triangle *b* obtained by Equation (2) are in Table 2. In the Equations (1) and (2) *Δz* is stepdown (vertical step); *R*_RT_ is the radius of the SPIF forming tool; *t* is time; *v*_f_ is feed rate; and *f* is frequency. However, the influential length of the bending during the SPIF process of PA sheets could not be considered as the length *b* only. Therefore, three times the length *b* was selected as the influential length used for frequency calculations of the DMA test.

After the calculation of inner angle *β* and side *b*, the next step was the determination of time *t* that the tool needed to pass the impact contact zone in the SPIF process. According to the feed rates of the SPIF process used by Martins and Marques [57,58] for the calculation of conditions at DMA three-point bending, values of *v*_f1_ = 1000 mm/min, *v*_f2_ = 1500 mm/min, and *v*_f3_ = 2000 mm/min were selected. The calculated times *t* (Equation (3)) for all three federates at various process parameters are presented in Table 3.

(3) $$t=\frac{3b}{v_{f}}$$

Finally, a frequency range necessary for the DMA three-point bending test was defined for the various parameters of the SPIF process. The results of frequency obtained with Equation (4) for particular combinations of process parameters are evident from Table 4.

(4) $$f=\frac{1}{t}.$$

From the obtained results (Table 4), it was apparent that the minimum loading–unloading frequency that was expected to occur during the SPIF process was 2.23 Hz, and the maximum was 9.01 Hz. According to the obtained data, it was advisable to perform DMA three-point bending tests at three different frequencies. In the first DMA three-point bending test, the frequency of 1 Hz was selected as it was below the minimal value presented in Table 4; the second selected frequency was 5 Hz, while the third frequency of 10 Hz was above the maximal frequency presented in Table 4.

## 3. Results and Discussion

### 3.1. Structure and Morphology

To determine the structure and morphology of the obtained nanocomposites, the XRD and TEM methods were used. XRD analysis is one of the most common methods for investigating the structure of polymeric nanocomposites [80]. Since the structure and morphology of nanocomposites cannot be reliably determined by one method, the TEM evaluation was also used. 

**Figure 3:**
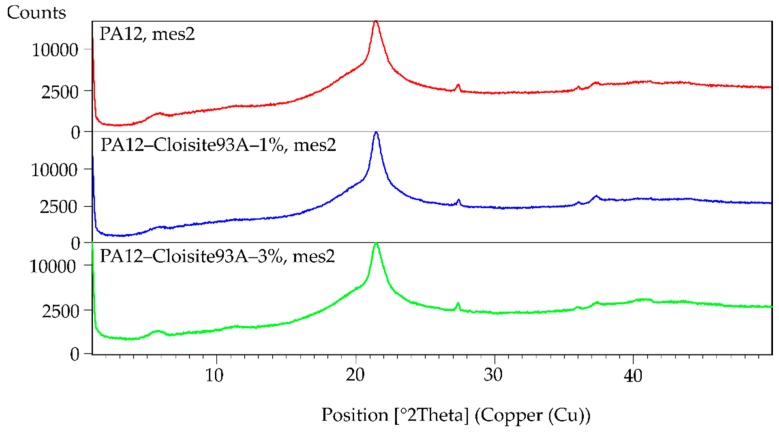
XRD graphs of PA12, PA12/1% of Cloisite 93A and PA12/3% of Cloisite 93A.

**Figure 4:**
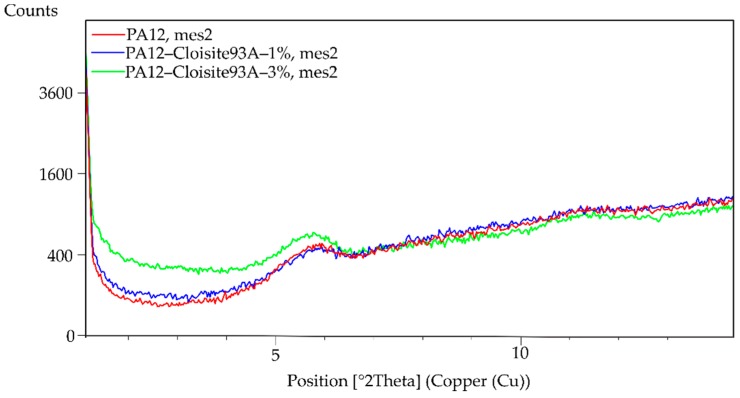
XRD graph (detail) of PA12, PA12/1% of Cloisite 93A and PA12/3% of Cloisite 93A.

The XRD patterns showed the absence of basal reflection d_001_, as is evident from Figure 3 and Figure 4. This fact could indicate that the nanocomposite structure was exfoliated. However, XRD results may give a false result depending on the orientation of the filler in the analysed sample of the polymer matrix. For this reason, the material was measured in different directions, always obtaining the same results. No peak on the diagram connected with the filler was observed. Despite this fact, the XRD was combined with another characterisation method. In this case, the prepared samples were evaluated using TEM.

**Figure 5:**
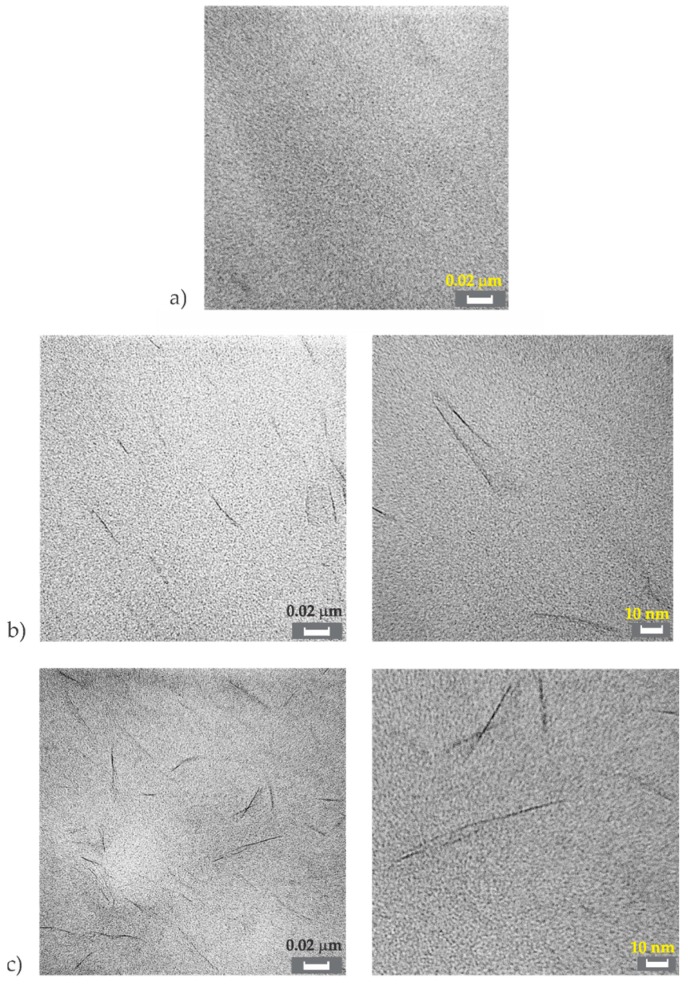
TEM micrographs of (**a**) PA12, (**b**) PA12 nanocomposite with 1% clay and (**c**) PA12 nanocomposite with 3% clay.

TEM pictures of neat PA12 and PA12 nanocomposites are presented in Figure 5. Figure 5a represents neat PA12. It was apparent that the structure was homogeneous in this case.

Figure 5b shows the structure of composite with 1% of clay at different magnifications. The dark lines represent the Cloisite 93A nano-filler, for which a good dispersion of the filler inside the PA polymer matrix was evident. It was also possible to see the stacks of several layers (Figure 5b—right). Based on these figures and XRD pattern, it could be concluded that a good level of exfoliation was reached. Exfoliated morphology is desired because of the large contact area between polymer matrix and filler, resulting in optimal material properties [81].

Figure 5c shows the structure of composite with the highest analysed filler content with 3% of clay. This composition (Figure 5c—left) also showed good morphology with a homogeneous matrix and uniformly distributed filler layers. Despite the increase of the filler content, there was no formation of agglomerate observed in the analysed composition. The structure may have contained the exfoliated layers together with stacks of several layers, probably two to three layers in stacks (Figure 5c—right). However, the morphology for both compositions with 1% and 3% on clay filler looked very similar.

It has to be mentioned that complete exfoliation of clays in the polymer matrix is not easy to achieve. However, most of the polymer nanocomposites reported in the literature possess less desirable intercalated structures [81]. Based on obtained results from XRD and TEM analysis, it was concluded that the processing of PA12/Cloisite 93A nanocomposites was successful.

The material phase transition temperature, like the melting temperature, *T_m_*, glass transition temperature, *T*_g_, crystallization temperature, *T_cc_*, crystallinity, *X*, were evaluated with DSC measurement. For determination of crystallinity, *X,* Equation (5) was used. In Equation (5) Δ*H*_f, norm_ is the normalised melting enthalpy, obtained from the DSC device with respect to the weight fraction of the corresponding component; *ΔH*_f100_ is the melting enthalpy of 100% crystalline PA12 with a value of 95 J/g [82]. Next, the samples were evaluated with DSC to determine the phase transition temperature and crystallinity. The results are presented in Table 5. The differences for *T*_g_ and *T*_m_ were up to 5%. The crystallinity composites PA12 with Cloisite 93A changed from 7% to 17% in comparison to pure PA12. This fact could be connected with the crystal size. With higher loading, the number of nucleation places should grow, and this results in a finer structure of the material. Such material then requires less heat for complete melting. The data confirmed the findings from the XRD and TEM analyses.

(5) $$X=\left(\frac{\Delta H_{f,\;\mathrm{norm}}}{\Delta H_{f100}}\right)\cdot 100$$

### 3.2. Mechanical Properties

#### 3.2.1. Tensile Test

The most common test for the characterisation of a material’s mechanical properties is the tensile test. In this study, the mechanical properties were tested for neat PA12 as well as for nanocomposites with 1% and 3% clay. 

In accordance with standard EN ISO 527-3, attention was given to tensile strength (*σ*_m_), Young’s modulus (*E*) and elongation (*ε*). The obtained data are in Table 6. The neat material PA12 showed the value of *E*-modulus of 875 MPa. The *E*-modulus of nanocomposites with 1% clay was 12% lower in comparison to the neat PA12. For PA12/Cloisite 93A–3%, moderate growth of the *E*-modulus was observed (+4%). In the case of tensile strength, the neat PA12 expressed a value of 52 MPa while for the PA12/Cloisite 93A–1% it was 54 MPa, and for PA12/Cloisite 93A–3% it was 55 MPa. It could be concluded that a further increase in filler content from 1% to 3% was not followed by a significant increase in tensile strength. The differences among the neat material and PA12/Cloisite 93A with different filler amounts are seen in Table 7. Regarding elongation at the break, a significant increase (more than 40%) was observed for PA12/93A composites, in comparison to the neat PA12. From these results, it was evident that the material did not strengthen significantly due to the amount of clay filler in the tested range. However, the addition of clay filler drastically increased the formability of the analysed nanocomposite, which is crucial for its successful implementation in SPIF technology.

In addition to the tensile tests used for the determination of the material’s mechanical properties, DMA was performed. Recently, the DMA method has become crucial when testing the properties of functional composites [83]. For the research of nanocomposite material oriented towards the determination of properties needed for the SPIF process, the DMA tensile test, as well as DMA three-point bending test, were also performed. 

**Figure 6:**
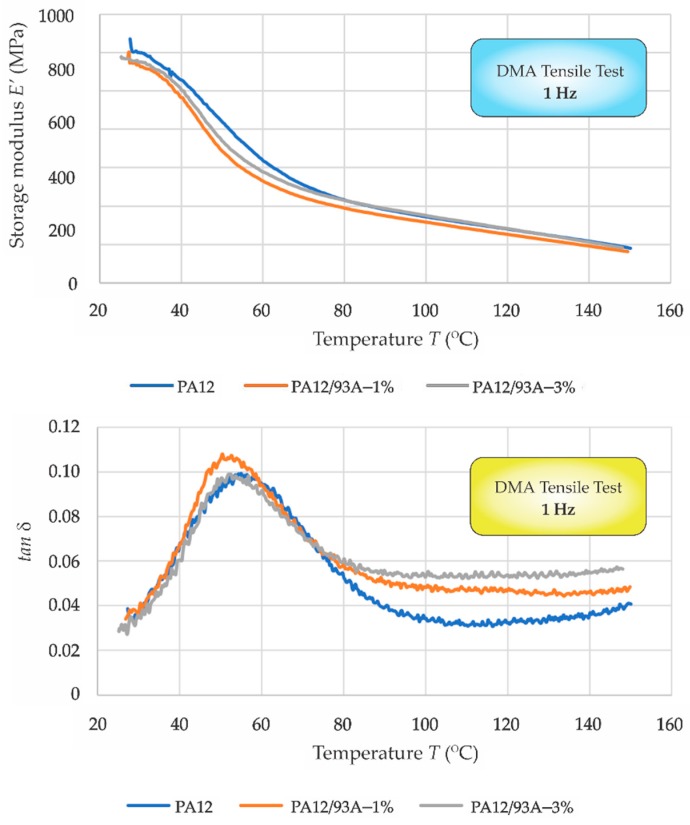
Dynamic mechanical analysis (DMA)—tensile 1 Hz: (**top**) storage modulus (*E’*) versus temperature (*T*) and (**bottom**) tan *δ* versus temperature (*T*).

From the results of the DMA tensile test, the glass transition temperature (*T*_g_) was characterised; see Figure 6. Determining the *T*_g_ can be done in several ways with the DMA technique. *T*_g_ is visible as a large drop in the storage modulus (*E’*) when viewed on the log scale versus a linear temperature scale [84]. The value reported as *T*_g_ is defined with the onset of *E’* drop; the peak of the tan *δ*, and the peak of the *E’* curve are the most commonly used [84]. Therefore, the peak of tan *δ* is determined as *T*_g_ (Figure 6 bottom). The following values of *T*_g_ were set: neat PA12 55 °C, PA12/Cloisite 93A–1% 51 °C and PA12/Cloisite 93A–3% 52 °C. The *T*_g_ were very similar for all compositions; the difference was in the range of 5%–7 %. Here, the obtained values of *T*_g_ were slightly different than those obtained with the DSC method. The determination of *T*_g_ by DMA in the presented case obtained from the diagrams and the method seemed to be less accurate than the DSC method. However, a slight decrease of *T*_g_ in the case of PA12/Cloisite 93A–1% can be observed in both cases and may have a positive influence on the SPIF process. 

#### 3.2.2. Three-Point Bending Test

**Figure 7:**
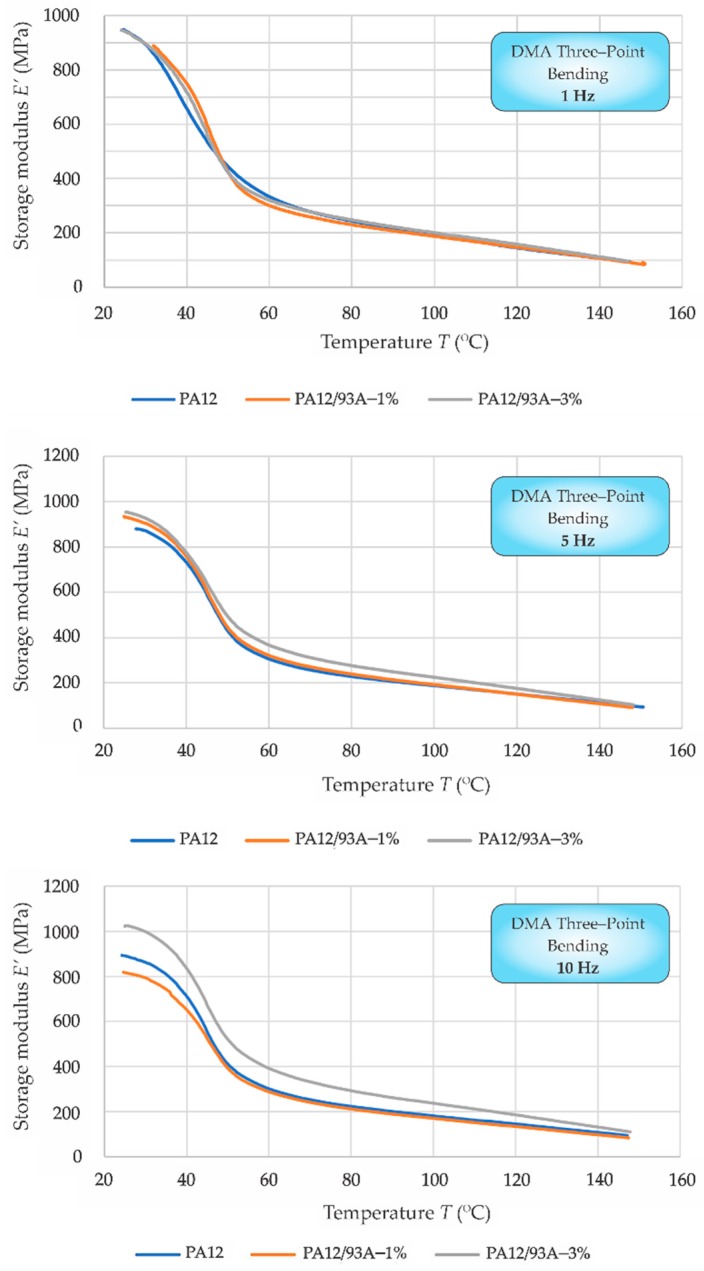
DMA three-point bending: storage modulus (*E’*) versus temperature (*T*) at 1 Hz (**top**), 5 Hz (**middle**) and 10 Hz (**bottom**).

DMA three-point bending mode was used due at conditions very close to the realistic ones, which occur during the SPIF process. The above conditions were calculated with the help of the terms given in Section 2.4. Figure 7 shows the storage modulus, *E’*, for all three testing frequencies; 1 Hz, 5 Hz, and 10 Hz respectively. 

Firstly, at the top of Figure 7, the first testing frequency of 1 Hz is presented. Very similar behaviour for mixtures with 1% and 3% of Cloisite 93A was noted. The prepared composites had slightly higher storage modulus, *E’*, in comparison to neat PA12 at lower temperatures. All the tested materials exhibited the same behaviour above 80 °C. The middle part of Figure 7 presents the evaluation of three-point bending DMA for the testing frequency of 5 Hz. Also, in this case, the storage modulus, *E’*, was slightly higher at lower temperatures in comparison to neat PA12. A weak increase of *E’* was noted for PA12/Cloisite 93A–3% from 45 °C onwards, expressing this deviation up to 140 °C. The behaviour of PA12/Cloisite 93A–1% is very similar to that of a neat PA12 material. Finally, the DMA results of storage modulus, *E’*, for a 10 Hz testing frequency are presented in Figure 7—bottom. The storage modulus of PA12/Cloisite 93A–3% is here clearly higher than the neat PA12 for the whole tested temperature range, with the largest difference of 14% at the lowest testing temperature of 25 °C. In contrast, the storage modulus, *E’*, of PA12/Cloisite 93A–1% was in the range from 25 °C to 50 °C lower than those of neat PA12 material. The highest difference can be observed at 25 °C, continuously decreasing towards 50 °C, at which point it finally disappeared. From 50 °C onwards, both materials had the same value of storage modulus.

**Figure 8:**
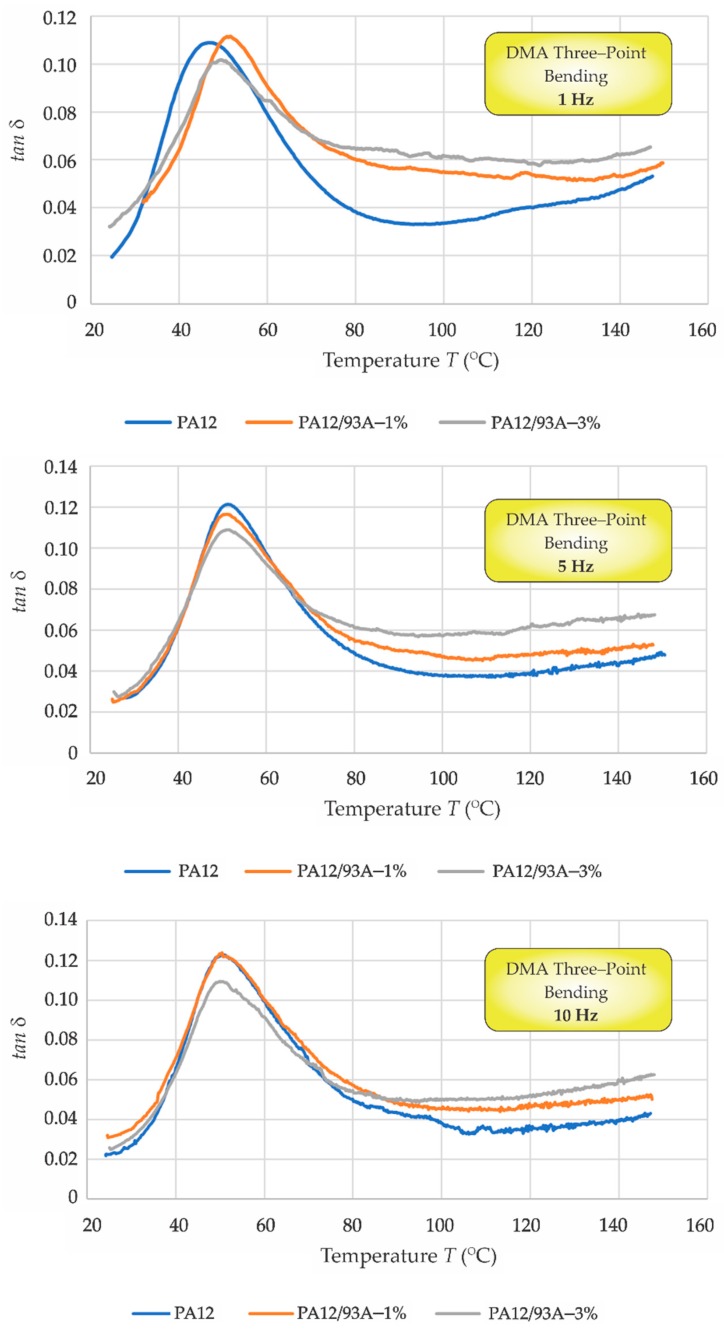
DMA three-point bending: tan *δ* versus temperature (*T*) at 1 Hz (**top**), 5 Hz (**middle**) and 10 Hz (**bottom**).

Figure 8 shows the loss tangent, tan *δ* for all three testing frequencies; 1 Hz (top), 5 Hz (middle) and 10 Hz (bottom), respectively. For all three testing frequencies, the loss tangent, tan *δ* for the pure PA12 and composites PA12/Cloisite 93A–1%, PA12/Cloisite 93A–3% was analysed. Furthermore, the testing at 1 Hz, the lowest frequency, was the same frequency also used for DMA tensile testing. The values of *T*_α_, which correlated with *T*_g_, are presented in Table 5. The highest value was the mixture with 1% of Cloisite 93A: the difference was only 6% in comparison to the neat PA12 material. However, this result was not in correlation with the data obtained from tensile mode and DSC, where the decrease of *T*_α_ and *T*_g_ was observed. These changes could be connected with the different geometries of the specimens used as well as different stress–strain conditions acting on the specimen during the DMA testing procedure. Furthermore, the filler dispersion and homogeneity of the material can play an important role. For the frequencies of 5 Hz and 10 Hz, similar values of *T*_α_ were detected for all tested materials.

From the DMA three-point bending results presented in Figure 7 it could be concluded, that changes in the mechanical behaviour of prepared compositions were not significant for PA12/Cloisite 93A–1%. Generally, it is known that adding nanoparticles into polymers increases their stiffness and decreases dissipation [85]. However, nanoparticles may directly interfere with the crystalline structures of semi-crystalline polymers [86,87] and stimulate the nucleation of growing crystals, influencing the size of growing crystals as well as the degree of crystallinity. With the increase of testing frequency, the composition PA12/Cloisite 93A–3% became stiffer in comparison to PA12/Cloisite 93A–1%. 

When comparing the obtained results of tensile and three-point bending tests conducted under the same testing conditions (frequency of 1 Hz, amplitude of 0.1 mm and ramp temperature of 2 °C/min), some discrepancies could be observed. The loading conditions were quite different for both tests. In the case of the tensile test, the material is stressed uniformly with pure tension across the entire cross-section. In case of the bending test, the specimen is stressed non-uniformly across its thickness. While on one surface the stresses are tensile, on the opposite surface compression loading appears. Furthermore, the crystalline structure of the surface layer differs from the crystalline structure of the inner layers due to the difference of material cooling during the solidification in the compression moulding of plate-shaped samples. Faster cooling on the surface can cause lower degrees of crystallinity compared to the inner layers and, consequently, in the lower stiffness of the outer layers of the samples. This lower stiffness cannot be expressed at tensile loading; however, it has significant influence on the bending test, in which the maximal stresses appears in the surface layers of the moulded specimen. The difference of both moduli can be attributed to the local/surface structural differences. Finally, it must be emphasized that the producer of the neat PA12 material reports on different values of flexural and tensile modulus [64], where the flexural modulus is, as in our case, lower.

For the selection of the proper nanocomposite that is the most suitable for SPIF technology, several aspects are to be taken into consideration. The main technological parameters of that SPIF process that also interact with the material selection are influenced by the tool movement. The forming process in SPIF is influenced by the tool path, the feed rate of the tool (which can additionally rotate around its vertical axis), step-down movement assuring the incremental part deformation, and tool geometry, for which the diameter plays the major role. The SPIF technology requires good formability of the material, which can be evaluated by the stretching of the material. The fact that polymers can be plastically deformed only above the *T*_g_ temperature, which plays a significant role in the selection of material applicable for SPIF, must be taken into consideration. Furthermore, it is to be expected that the high storage modulus, *E’*, has a negative influence on tool loads during the SPIF process. A similar influence is to be expected regarding the material tensile strength. Through this, the observation of materials’ properties analysed in the paper can be discussed as follows:

- Stretchability: Both nanocomposites expressed significantly better elongation at break in comparison to the neat PA12 material; however, their values were similar. More specifically, the PA12/Cloisite 93A–3% had 3% higher elongation at break and could be favoured regarding this parameter.
- Glass transition temperature *T*_g_: To obtain the formability of the polymer during the SPIF process at the lowest possible temperatures is desirable; therefore, the determination of the glass transition temperature is indispensable. Among all three analysed materials, the PA12/Cloisite 93A–1% had the lowest *T*_g_ at 57 °C and could be, according to this parameter, favoured for selection of SPIF technology.
- Storage modulus *E’*: Due to the local bending and unbending process, this parameter is of importance during the SPIF process. Its determination through DMA three-point bending showed that in the range applicable for SPIF (above the *T*_g_) it was distinguished at the conditions at lower and higher bending frequencies. At lower frequencies of 1 Hz and 5 Hz, all materials expressed similar values of *E’*. However, at the highest analysed frequency of 10 Hz, the PA12/Cloisite 93A–3% expresser noticeably higher values of *E’* than the other two materials. Since, due to the shortening of production times at SPIF, the higher feed rates, also causing higher bending frequencies, are preferred, and the PA12/Cloisite 93A–3% material was not favourable in this case.

**Figure 9:**
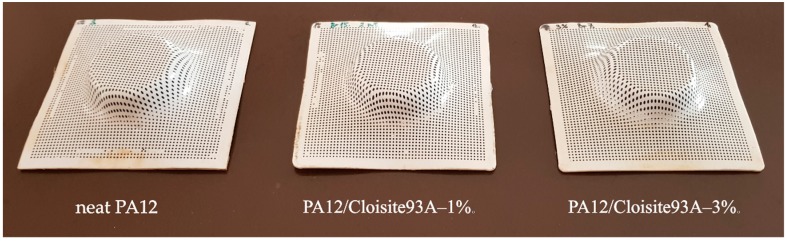
Test parts obtained with SPIF technology: PA12 (**left**), PA12 nanocomposite with 1% clay (**middle**) and PA12 nanocomposite with 3% clay (**right**).

In summarizing all the above-stated influences of material parameters on forming by SPIF, the PA12/Cloisite 93A–1% could be selected as a favourable material. Preliminary SPIF tests with neat PA12 and selected PA12 composites were carried out for first evaluations of the materials’ formability (Figure 9). In this stage, we proved the applicability of all selected materials for SPIF technology. However, further research work should be performed regarding the interactions of particular technological parameters (feed rate, drawing angle, step size, tool radius, etc.) with the specific material properties of all three materials obtained from DMA material response, DSC analysis, and the measured values of mechanical properties (Table 5). Finally, it is necessary to define the optimal parameter combination of SPIF technology for each of the analysed PA12 materials.

## 4. Conclusions 

In this paper, the preparation and characterisation of polymer materials that are potentially suitable for single point incremental forming technology (SPIF) are described. The PA12 polymer group was selected due to the high material strength, which is sought for some cases of SPIF-produced parts. The tested materials were neat PA12 and nanocomposites PA12/Cloisite 93A–1% and PA12/Cloisite 93A–3%. The structure and morphology, together with mechanical properties being predominant for SPIF, were examined in this study. The main goal was to find the optimal composition of analysed nanocomposites adopted for further processing by SPIF. Preliminary tests to verify the applicability of selected materials for SPIF technology were already conducted.

On the base of XRD and TEM results, it was concluded that a good level of exfoliation of clay in the polymer matrix was reached. There was no agglomeration in compositions with clay Cloisite 93A. Furthermore, the DSC evaluation was in correlation with XRD and TEM results. Therefore, it can be concluded that during the mixing and preparation of nanocomposites, the proper processing conditions were selected.

The filler content in the PA12 matrix showed a direct impact on the mechanical properties of the obtained nanocomposites. From the obtained results, it was apparent that the analysed nanocomposites with 1% and 3% Cloisite 93A expressed 4% and 6% higher tensile strength, respectively, in comparison to neat PA12. The *E*-modulus was decreased by 12% when 1% of Cloisite 93A was used as the filler in nanocomposite while this change at 3% of Cloisite 93A in the nanocomposite was slightly higher, with an increase of 4%.

Analysing the flexural and tensile modulus of the observed PA12 materials revealed differences among both parameters. Despite the fact that both tests were performed under the same testing conditions, the stress state was rather different in both cases. Regarding lower flexural strength, the most stressed surface layers may have expressed a larger influence on *E’* due to non-uniform material solidification at compression moulding, as in the case of tensile modulus.

For SPIF technology, it is desirable that the material provide high elongation. In a comparison of the material’s elongation to the neat PA12, it was determined that the mixture PA12/Cloisite 93A–1% expressed an increase of 44% and the mixture PA12/Cloisite 93A–3% an increase of 49%. 

The DSC analysis showed the lowest value of *T*_g_ at 57 °C for PA12/Cloisite 93A–1% and was therefore favourable with regard to the start of material’s plastic deformation at the SPIF process. Finally, the DMA test delivered improved properties of the composition PA12/Cloisite 93A–1% regarding the *T*_α_, which dropped by 5%. Furthermore, a slight drop of *T*_α_ of 2% can be observed for PA12/Cloisite 93A–3%. Measured melting points *T_m_* are less relevant for the SPIF technology because such high workpiece temperatures during the processing are not allowed. In comparison to the neat material the PA12/Cloisite 93A–1% was not sensitive on bending frequency above the temperature *T*_α_ while the PA12/Cloisite 93A–3% expressed increase of storage modulus *E’* at 10 Hz testing frequency in comparison to other two materials.

In comparison to the other two analysed materials, the composition of PA12/Cloisite 93A–1% showed better properties as lower *T*_g_ and *T*_α_ combined with high material stretchability without an increase of storage modulus *E’* in comparison to the neat PA12 at high testing frequencies of the three-point bending mode. Currently, it seems that this composition is favourable for analyses of further interactions with the technological parameters of SPIF technology.

## Figures and Tables

**Table 1:** The main technical features of the twin screw counter-rotating Brabender extruder.

***D***	42 mm
***L***	252 mm
***L*** **/** ***D***	6
**Max. Temperature**	350 °C

**Table 2:** The results for the inner angle of spherical triangle *β* and side of spherical triangle *b* obtained from Equations (1) and (2).

Index	**“11”** *Δz*_1_ = 0.25 mm *R_RT_*_1_ = 3 mm	**“12”** *Δz*_2_ = 0.25 mm *R_RT_*_1_ = 3 mm	**“13”** *Δz*_3_ = 0.25 mm *R_RT_*_1_ = 3 mm	**“21”** *Δz*_1_ = 0.25 mm *R_RT_*_2_ = 4 mm	**“22”** *Δz*_2_ = 0.25 mm *R_RT_*_2_ = 4 mm	**“23”** *Δz*_3_ = 0.25 mm *R_RT_*_2_ = 4 mm
*β* (rad)	0.411	0.586	0.723	0.355	0.505	0.622
*b* (mm)	1.233	1.757	2.168	1.422	2.021	2.489

**Table 3:** The results for time obtained from Equation (3) expressed in seconds (s).

Technological Parameters	*v*_f1_ = 1000 mm/min = 16.67 mm/s A	*v*_f2_ = 1500 mm/min = 25 mm/s B	*v*_f3_ = 2000 mm/min = 33.33 mm/s C
*t*_11_	0.222	0.148	0.111
*t*_12_	0.316	0.211	0.158
*t*_13_	0.390	0.260	0.195
*t*_2__1_	0.256	0.171	0.128
*t*_22_	0.364	0.243	0.182
*t*_23_	0.448	0.299	0.224

**Table 4:** The results for frequency obtained from Equation (4).

Condition	A [Hz]	B [Hz]	C [Hz]
*f*_11_	4.50	6.76	9.01
*f*_12_	3.16	4.74	6.32
*f*_13_	2.56	3.84	5.12
*f*_21_	3.91	5.86	7.82
*f*_22_	2.75	4.12	5.50
*f*_23_	2.23	3.35	4.46

**Table 5:** Differential scanning calorimetry (DSC) evaluation.

	PA12	PA12/Cloisite 93A–1%	PA12/Cloisite 93A –3%
*T*_g_ (°C)	60	57	58
*T_m_* (°C)	179	179	183
*T_cc_* (°C)	150	150	150
*X* (%)	69	64	57
*T*_α_ tensile (°C)	54	51	53
*T*_α_ bending 1 Hz (°C)	48	52	50
*T*_α_ bending 5 Hz (°C)	51	51	51
*T*_α_ bending 10 Hz (°C)	51	51	50

**Table 6:** The average values with standard deviations in brackets of mechanical properties for PA12 and nanocomposites.

	PA12	PA12/Cloisite 93A–1%	PA12/Cloisite 93A–3%
*E* [MPa]	875 (± 35)	771 (± 63)	913 (± 62)
*σ*_m_ [MPa]	52 (± 3)	54 (± 1)	55 (± 3)
*ε* [%]	45 (± 11)	65 (± 12)	67 (± 7)

**Table 7:** Comparison of relative average values of mechanical properties between PA12 and nanocomposites.

	PA12	PA12/Cloisite 93A–1%	PA12/Cloisite 93A–3%
*Rel E* [1]	1	–12%	4%
*Rel σ*_m_ [1]	1	4%	6%
*Rel ε* [1]	1	44%	49%
